# Characterization and Application of Enterocin RM6, a Bacteriocin from *Enterococcus faecalis*

**DOI:** 10.1155/2013/206917

**Published:** 2013-06-13

**Authors:** En Huang, Liwen Zhang, Yoon-Kyung Chung, Zuoxing Zheng, Ahmed E. Yousef

**Affiliations:** ^1^Department of Food Science and Technology, The Ohio State University, Columbus, OH 43210, USA; ^2^Mass Spectrometry and Proteomics Facility, The Ohio State University, Columbus, OH 43210, USA; ^3^Department of Food & Biotechnology, Hankyong National University, Gyeonggi 456749, Republic of Korea; ^4^Central Research, Kraft Foods Group, Inc., Glenview, IL 60025, USA; ^5^Department of Microbiology, The Ohio State University, Columbus, OH 43210, USA

## Abstract

Use of bacteriocins in food preservation has received great attention in recent years. The goal of this study is to characterize enterocin RM6 from *Enterococcus faecalis* OSY-RM6 and investigate its efficacy against *Listeria monocytogenes* in cottage cheese. Enterocin RM6 was purified from *E. faecalis* culture supernatant using ion exchange column, multiple C_18_-silica cartridges, followed by reverse-phase high-performance liquid chromatography. The molecular weight of enterocin RM6 is 7145.0823 as determined by mass spectrometry (MS). Tandem mass spectrometry (MS/MS) analysis revealed that enterocin RM6 is a 70-residue cyclic peptide with a head-to-tail linkage between methionine and tryptophan residues. The peptide sequence of enterocin RM6 was further confirmed by sequencing the structural gene of the peptide. Enterocin RM6 is active against Gram-positive bacteria, including *L. monocytogenes*, *Bacillus cereus,* and methicillin-resistant *Staphylococcus aureus* (MRSA). Enterocin RM6 (final concentration in cottage cheese, 80 AU/mL) caused a 4-log reduction in population of *L. monocytogenes* inoculated in cottage cheese within 30 min of treatment. Therefore, enterocin RM6 has potential applications as a potent antimicrobial peptide against foodborne pathogens in food.

## 1. Introduction

*Listeria monocytogenes* is the causative agent of human listeriosis, a disease with 20–30% fatality [1]. *L. monocytogenes* is an ubiquitous microorganism that can be found in many raw and processed foods. Soft cheese products have been associated with several listeriosis outbreaks [2–4]. Rudolf and Scherer [5] reported that *L. monocytogenes *was found in 6.4% of European red smear cheese. Recently, a multistate listeriosis outbreak occurred in the United States due to consumption of contaminated cantaloupe, resulting in 30 deaths across 28 states [6]. Therefore, control of *L. monocytogenes* remains an important issue for the food industry.

Use of bacteriocins in food preservation has gained a great attention in recent years [7]. The effect of many antilisterial bacteriocins (e.g., nisin, pediocin, and enterocin AS-48) has been investigated in foods including dairy products, meats, and fresh produce [8–10]. In this study, we described a new isolate of *Enterococcus faecalis *from raw milk, which produces an anti-listerial peptide, enterocin RM6. The bacteriocin was purified by liquid chromatography techniques. The peptide sequence was determined by mass spectrometry and confirmed by structural gene sequencing. In addition, the efficacy of enterocin RM6 against *L. monocytogenes* in cottage cheese was investigated.

## 2. Materials and Methods

### 2.1. Bacterial Strains and Media

Strains were obtained from the culture collection of the food safety laboratory at The Ohio State University (Columbus, OH, USA). The producer strain, *E. faecalis *OSY-RM6, which was isolated from raw milk [11], was grown in MRS broth (Becton and Dickinson, Sparks, MD, USA). The indicator strain, *Lactobacillus cellobiosus* OSU 919, was cultivated in MRS broth. Other bacterial strains used in the test of antimicrobial spectrum are listed in Table 1.

### 2.2. Strain Identification by 16S rDNA Sequencing

Genomic DNA of strain OSY-RM6 was purified using a DNA isolation kit (DNeasy Blood & Tissue kit; QIAGEN, Valencia, CA, USA). The 16S rDNA sequence was amplified by PCR using two universal primers specific for rDNA gene [12]. PCR amplification was performed using a *Taq *DNA polymerase kit (QIAGEN) under the following conditions: the reaction mixture was subjected to an initial denaturation at 94°C for 3 min, followed by 35 cycles, including 1 min at 94°C, 1 min at 52°C, and 2 min at 72°C. A final extension was carried out at 72°C for 10 min. The amplified PCR product was purified using a gel extraction kit (QIAquick, QIAGEN), ligated to the pGEM-T Easy vector (Promega, Madison, WI, USA), and introduced into competent *E. coli* DH5*α* cells by electroporation. The recombinant plasmid carrying the 16S rDNA fragment was isolated from overnight culture of *E. coli* using spin column (QIAprep Spin Miniprep kit, QIAGEN). Resultant plasmid DNA was sequenced using a 3730 DNA Analyzer (Applied Biosystems, Foster city, CA, USA) at the Plant-Microbe Genomics Facility at The Ohio State University.

### 2.3. Purification of Enterocin RM6 from Cultured Broth

An aliquot of 0.5 mL of OSY-RM6 overnight culture was inoculated into a 1-liter flask containing 500 mL MRS broth. The flask was incubated at 30°C for 18 hours without shaking. Cells in the cultured broth were removed by centrifugation at 15,180 ×g for 15 min at 4°C (Sorvall RC-5B, DuPont, Wilmington, DE, USA). The supernatant was adjusted to pH 6.5 using 1.0 N NaOH and passed through a cationic exchange column (Macro-Prep High S support; Bio-Rad, Hercules, CA, USA) that was equilibrated with phosphate buffer (50 mM, pH 6.5). Enterocin RM6 retained on the column was eluted with 1.0 M NaCl in phosphate buffer (50 mM, pH 6.5). The resultant eluate was subjected to solid phase extraction using ten C_18_ silica cartridges (Sep-Pak; Waters Corporation, Milford, MA, USA) that were head-to-tail connected to increase the binding capacity. Enterocin RM6 was eluted from the cartridges by 70% acetonitrile, and the solvents were removed by lyophilization. The crude extract (CE) of enterocin was obtained by dissolving the freeze-dried powder in high-performance liquid chromatography (HPLC) grade water.

Crude extract of enterocin was further purified by reverse-phase HPLC (Hewlett Packard 1050, Agilent Technologies, Palo Alto, CA, USA). Separation was achieved using a preparative column with 5 *μ*m particle size (250 mm × 10 mm; Alltech Associates, Inc., Deerfield, IL, USA). The mobile phase consisted of (A) a mixture of isopropanol and acetonitrile (2 : 1, v/v) with 0.4% trifluoroacetic acid (TFA) and (B) HPLC grade water with 0.1% TFA. For each run, aliquots (300 *μ*L) of crude extract were loaded and separated on the column by a linear gradient of 0 to 100% solvent A over 30 min, followed by 100% solvent A for 5 min at a flow rate of 1.5 mL/min. Elution was monitored using a UV-detector at a wavelength of 280 nm. Fractions from each minute were collected automatically using Waters Fraction Collector II (Waters Cooperation, Milford, MA, USA). Fractions with the same retention time from multiple runs were pooled and lyophilized; the resulting powder was dissolved in water. Antimicrobial activity of each faction was determined by microtiter plate bioassay. Active fraction against bacterial indicator strain was stored at 4°C for further analyses.

### 2.4. Antimicrobial Activity Determination and Inhibition Spectrum

Antimicrobial activity was determined as described by Yousef and Carlstrom [13]. *L. cellobiosus* OSU 919 was used as the indicator. Briefly, aliquots (10 *µ*L) of overnight indicator bacterium were transferred into 9 mL of MRS soft agar (0.75%) that was held at ~50°C; the mixture was then poured onto a basal MRS agar plate. The tested compound or solution with enterocin RM6 was subjected to serial two-fold dilutions. Aliquots (5 *µ*L) of diluted solution were spotted onto the soft agar layer seeded with indicator bacterium. After incubation at 30°C overnight, inhibitory areas were observed. Antimicrobial activity is expressed in arbitrary unit (AU/mL), which is the reciprocal of the highest dilution factor resulting in a clear inhibitory zone.

Antimicrobial activity of HPLC fractions was examined by microtiter plate bioassay. Briefly, overnight indicator culture was tenfold diluted using MRS medium, and 100 *µ*L of diluted cell suspension was added in a 96-well plate. An equal volume (100 *µ*L) of HPLC fraction was added to each well and incubated at 30°C for 5–8 hours, where sterile distilled water was used as negative control. Optical density at 600 nm (OD_600_) was measured using a spectrophotometric microplate reader (Vmax Kinetic Microplate Reader, Molecular Devices Corp., Menlo Park, CA, USA). Active fraction showed a lower OD_600_ value compared to the negative control because of the inhibitory effect against the indicator bacterium.

### 2.5. ESI-MS and MS/MS Analyses

Accurate molecular weight determination and further peptide sequence investigation of enterocin RM6 were performed on a mass spectrometer (LTQ orbitrap, ThermoFinnigan, West Palm Beach, FL, USA) operated in positive ion mode. Briefly, the sample diluted in the mixture of H_2_O-MeOH-HAc (50 : 50 : 2.5) was infused into the electrospray source at a 6 *µ*L/min flow rate. To achieve the optimal electrospray, spray voltage was set at 2,000 V; source temperature was 175°C. The data were recorded between 400 and 2000 Da, and the resolution was set at 30000 to achieve high mass accuracy determination. The most abundant enterocin RM6 peak was isolated for further MS/MS study. The isolation window was set at 10 Da and the CID fragmentation energy was set to 35%. Data were acquired in continuum mode until well-averaged data were obtained.

### 2.6. Structural Gene of Enterocin RM6

Genomic DNA from strain OSY-RM6 was used as template for amplifying the structural gene. Two primers (Ent48SF: 5′-GAGGAGTITCATGITTAAAGA-3′ and Ent48SR: 5′-CATATTGTTAAATTACCAAGCAA-3′) were used for PCR [14]. PCR amplification was performed using a *Taq *DNA polymerase kit (QIAGEN) under the following conditions: the reaction mixture was subjected to an initial denaturation at 95°C for 5 min, followed by 30 cycles, including 1 min at 95°C, 1 min at 52°C, and 1 min at 72°C. A final extension was carried out at 72°C for 10 min. The resultant PCR product was cloned in pGEM-T Easy vector for DNA sequencing.

### 2.7. Efficacy of Enterocin RM6 against *L. monocytogenes* in Inoculated Cottage Cheese

Twenty gram of cottage cheese (2% reduced milk fact, Kraft foods) was mixed with 30 mL of peptone water (0.1%), and the mixture was homogenized in a stomacher for 3 minutes. Enterocin crude extract was added to the diluted cheese sample at a final concentration of 80 AU/mL. Peptone water (0.1%) was used as negative control. The mixture was inoculated with *L. monocytogenes* Scott A at a final concentration of ~10^5^ CFU/mL. Inoculated cheese samples were incubated at 35°C for 26 hours. Samples were analyzed at 0.5 h, 4 h, and 26 hours after treatment to examine the inhibitory effect of enterocin. Viable cells after treatment were counted using tryptic soy agar, PALCAM agar, and modified Oxford agar (Becton and Dickinson). Each treatment or control included two independent experiments.

## 3. Results

### 3.1. Strain Identification

The producer strain OSY-RM6 is a Gram-positive bacterium. Analysis of the 16S rDNA gene (accession number: KF154976) showed a 99% similarity to *E. faecalis. *The new isolate was designated as *E. faecalis* OSY-RM6. This strain was positive for acid production (pH 4.8) in MRS broth and coagulated milk protein within 24 hours.

### 3.2. Purification of Enterocin RM6

**Figure 1:**
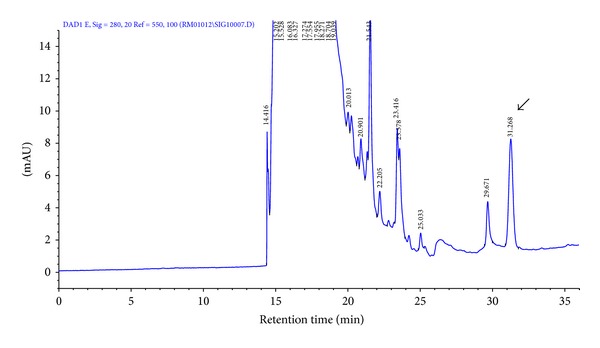
High-performance liquid chromatography profile of the crude extract of enterocin RM6. The peak with retention time of 31.27 min (indicated by the arrow) showed antimicrobial activity against *Lactobacillus cellobiosus* OSU 919.

 The procedure for preparing enterocin RM6 crude extract recovered ca. 24% total activity in the culture broth (Table 1). The crude extract was further purified by reverse-phase HPLC using a preparative column. The retention time of enterocin RM6 on a C_18_ column was 31.268 min (Figure 1).

### 3.3. Molecular Weight and Peptide Sequence Determination by MS and MS/MS

**Figure 2:**
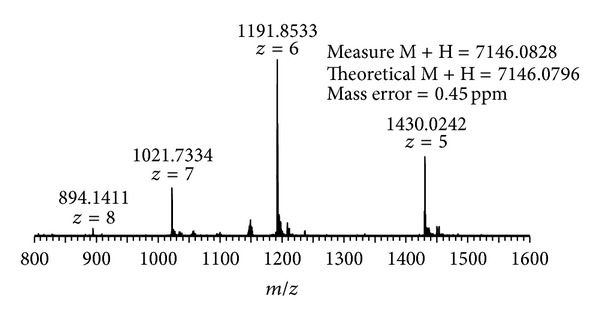
Electrospray ionization-mass spectrometry (ESI-MS) analysis of enterocin RM6.

**Figure 3:**
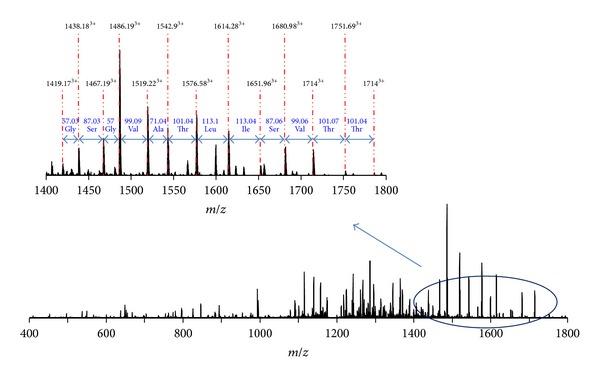
Fragmentation of enterocin RM6 examined by tandem mass spectrometry (MS/MS).

**Figure 4:**
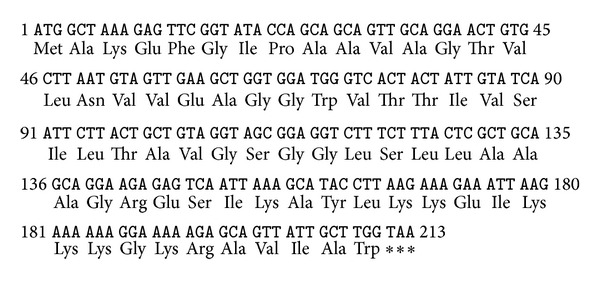
Structural gene of enterocin RM6 and the deduced peptide sequence.

HPLC-purified enterocin RM6 was subjected to electrospray ionization mass spectrometry (ESI-MS) analysis for accurate molecular weight determination. Additional MS/MS analysis was also performed to obtain the amino acid sequence of enterocin RM6. As shown in Figure 2, peaks carrying different charge status (*m*/*z* = 894.1411^8+^, 1021.7334^7+^, 1191.8533^6+^, and 1430.0242^5+^) were observed for intact enterocin RM6. After deconvolution, the average monoisotopic mass (M + H) of enterocin RM6 was calculated as 7146.0828 Da. The most abundant peak (*m*/*z* = 1191.8533^6+^) was then isolated and further fragmented by collision-induced dissociation (CID) to obtain the sequential information of this peptide. A series of MS/MS product ions were observed at *m*/*z* = 1419.17^3+^, 1438.18^3+^, 1467.19^3+^, 1486.19^3+^, 1519.22^3+^, 1542.90^3+^, 1576.58^3+^, 1614.28^3+^, and 1680.98^3+^, which led to the identification of a fragment with 12 amino acids: IVSILTAVGSGG or GGSGVATLISVI (note that L and I can be switched since they have identical masses) (Figure 3). This 12 amino acids sequence partially matches the sequence of a 70-residue cyclic peptide: AS-48 protein, whose complete sequence is MAKEFGIPAAVAGTVLNVVEAGGWVTTIVSILTAVG SGGLSLLAAAGRESIKAYLKKEIKKKGKRAVIAW, where a tail-to-head linkage is formed between the N-terminal methionine and the C-terminal tryptophan through the dehydration of one water molecule [14, 15]. The theoretical molecular weight of AS-48 protein is 7145.0718 Da, which matches exactly with the observed enterocin RM6 molecular weight (7145.0745 Da). Mass accuracy between theoretical and measured mass of enterocin RM6 is 0.44 ppm. In addition, more product ions were observed in the MS/MS spectrum (See Table S1 in Supplementary Material available online at http://dx.doi.org/10.1155/2013/206917) which further supported that enterocin RM6 shares the same sequence with peptide AS-48 [14, 15]. Fragment ions were observed at *m*/*z* = 575.32, 646.36, 777.40, 963.48, and 1034.51, which corresponded to sequences KEFGI, AKEFGI, MAKEFGI, WMAKEFGI, and AWMAKEFGI, respectively. This observation thus confirmed that the NH_2_ terminus in Met1 was linked with the COOH-terminus in Trp70 to form the head-to-tail cyclized peptide. During CID fragmentation, the cyclized peptide can be broken at different locations to generate different fragmentation patterns. For example, when the linkage was broken between Ile8 and Pro9, internal ions identified as PAAVA (*m*/*z* = 410.24), PAAVAG (*m*/*z* = 467.26), PAAVAGT (*m*/*z* = 568.31), PAAVAGTV (*m*/*z* = 667.38), and PAAVAGVTLNVVEAGGWVTTIV (*m*/*z* = 1053.59^2+^) were observed. Other fragment ions that were observed included ions that represent amino acids sequences KEFGI, AKEFGI, MAKEFGI, WMAKEFGI, and AWMAKEFGI, as well as other amino acid sequences between SLLAAAGRESIKAYLKKEIKKKGKRAVIAWMAKEFGI and EAGGWVTTIVSILTAVGSGGLSLLAAAGRESIKAYLKKEIKKKGKRAVIAWMAKEFGI. Observation of other product ions suggested that the cyclized peptide can also be broken between Ala11 and Val12, Val12 and Ala13, and Val19 and Val20. Detailed MS/MS fragment assignments are listed in Table S1. In addition, the peptide sequence was confirmed by determination of the structural gene of enterocin RM6 (accession number: KF154975). The deduced peptide sequence from structural gene agreed with the sequence determined by mass spectrometry (Figure 4).

### 3.4. Antimicrobial Spectrum of Enterocin

Enterocin RM6 is active against all tested Gram-positive bacteria, but it has no activity against Gram-negative bacteria (Table 2). *Pediococcus acidilactici* PO2 is the most sensitive strain to enterocin RM6. Moreover, this enterocin has strong activity against some important pathogens such as *L. monocytogenes* Scott A, *B. cereus* ATCC 14579, and methicillin-resistant *S. aureus* (MRSA).

### 3.5. Bactericidal Effect of Enterocin RM6 against Listeria in Cottage Cheese

**Figure 5:**
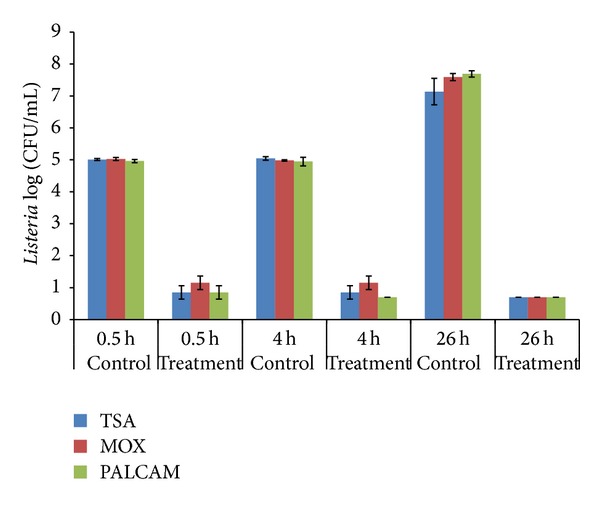
Effect of enterocin RM6 on survival of *L. monocytogenes *Scott A, inoculated on cottage cheese, using different media^a^.  ^a^TSA: tryptic soy agar; PALCAM: a selective media for *Listeria*; MOX: modified Oxford agar. Data represent the average of two independent experiments.

When enterocin crude extract (final concentration, 80 AU/mL) was added to *Listeria*-inoculated cottage cheese, a 4-log reduction in pathogen population was observed within 30 min and viable cells were not detected after 26 hrs. In contrast, the population of *L. monocytogenes* without treatment increased to 10^7^ CFU/mL after 26 hrs at the same incubation conditions (Figure 5).

## 4. Discussions

Bacteriocins are ribosomally synthesized antimicrobial peptides produced by some Gram-positive bacteria. Many bacteriocins from *Enterococcus *spp. have been purified and characterized, such as enterocin A, B, P, and AS-48 [16]. In this study, we describe a new bacterial strain isolated from raw milk exhibiting strong antimicrobial activity. The new isolate was identified as *E. faecalis* by 16S rDNA sequencing and was designated as strain OSY-RM6. MS/MS analyses and structural gene sequencing confirmed that the antimicrobial activity was attributed to a cyclic peptide with 70 residues, whose chemical structure is the same as enterocin AS-48.

The efficacy of bacteriocins is usually affected by conditions in the food ecosystems such as food composition [7]. The antilisterial efficacy of enterocin RM6 was investigated in cottage cheese. The results indicated that enterocin RM6 has a rapid bactericidal activity against *L. monocytogenes* in cheese products. In all, enterocin RM6 may have a practical application in the food industry to control listerial contamination.

## Supplementary Material

List of detailed daughter ions from tamdem mass spectrometry(MS/MS)analysis of enterocin RM6.

## Figures and Tables

**Table 1:** Preparation of crude extract of enterocin RM6.

Purification step	Volume (mL)	Arbitrary unit (AU/mL^a^)	Total activity (×10^3^ AU)	Recovery rate (%)
Culture supernatant	500	800	400	100
Cation exchange	75	1600	120	30
C_18_ silica cartridges	60	1600	96	24

^a^AU/mL: the reciprocal of the highest dilution factor showing a visible inhibitory zone.

**Table 2:** Antimicrobial spectrum of enterocin RM6.

Strains^a^	Media^b^	Diameter of inhibitory zone (mm)
Gram-positive bacteria		
* Pediococcus acidilactici *PO2	MRS	17.5
* Pediococcus pentosaceus *	MRS	16.8
* Lactobacillus plantarum *ATCC 8014	MRS	11.4
* Lactobacillus casei *ATCC 7469	MRS	13.4
* Lactobacillus acidophilus *ATCC 19992	MRS	10.0
* Pediococcus cerevisiae *	MRS	11.1
* Lactobacillus cellobiosus *OSU 919	MRS	14.8
* Listeria innocua *ATCC33090	TSBYE	7.4
* Enterococcus faecalis *ATCC 29212	MRS	8.4
* Listeria monocytogenes *Scott A	TSBYE	11.3
* Bacillus cereus *ATCC 14579	TSBYE	11.2
* Bacillus cereus *ATCC 11778	TSBYE	5.8
* Staphylococcus aureus *OSU 6538	NB	6.1
*Staphylococcus aureus* (methicillin sensitive)	NB	12.4
*Staphylococcus aureus* (methicillin resistant)	NA	10.0
Gram-negative bacteria		
* Yesinia enterocolitica *	TSBYE	—
* Salmonella Typhimurium *	LB	—
* Escherichia coli *O157:H7	LB	—

^a^Strains obtained from the culture collection of The Ohio State University food safety laboratory. ^b^MRS: lactobacillus MRS broth; TSBYE: tryptic soy broth supplemented with 0.6% yeast extract; NB: nutrient broth; LB: Luria-Bertani medium.
