# Supplementary material for: Characterization and Application of Enterocin RM6, a Bacteriocin from Enterococcus faecalis
Source: Biomed Res Int. 2013 Jun 13;2013:206917. doi: 10.1155/2013/206917 (PMC3697273; doi:10.1155/2013/206917)
Supplement: Supplementary file 1 — List of detailed daughter ions from tamdem mass spectrometry(MS/MS)analysis of enterocin RM6. [file 206917.f1.pdf]

Table S1 Detailed MSMS analysis of Enterocin RM6.

Theoretical m/z	Measured m/z	Δ Mass (Da)	Internal Ion Sequence
**Cyclized peptide broken in between I8 and P9**			
410.2398	410.24		PAAVA
467.2613	467.26	57.02 (Gly)	PAAVAG
568.3089	568.31	101.05 (Thr)	PAAVAGT
667.3774	667.38	99.07 (Val)	PAAVAGTV
780.4614	780.461	113.08 (Leu)	PAAVAGTVL
894.5043	894.501	114.04 (Asn)	PAAVAGTVLN
$993.5728/497.2903^{2+}$	$993.571/497.29^{2+}$	99.07 (Val)	PAAVAGTVLNV
$1092.6412/546.8245^{2+}$	$1092.64/546.82^{2+}$	99.07 (Val)	PAAVAGTVLNVV
$1221.6838/611.3458^{2+}$	$1221.68/611.34^{2+}$	129.04 (Glu)	PAAVAGTVLNVVE
$1292.7209/646.8644^{2+}$	$1292.72/646.86^{2+}$	71.04 (Ala)	PAAVAGTVLNVVEAG
$1406.7638/703.8858^{2+}$	$1406.76/703.88^{2+}$	114.04 (Gly-Gly)	PAAVAGTVLNVVEAGG
$1592.8431/796.9255^{2+}$	$1592.84/796.92^{2+}$	186.08 (Trp)	PAAVAGTVLNVVEAGGW
$1691.9115/846.4597^{2+}$	$1691.91/846.46^{2+}$	99.07 (Val)	PAAVAGTVLNVVEAGGWV
896.9835^{2+}	896.98^{2+}	101.04 (Thr)	PAAVAGTVLNVVEAGGWVT
1053.5840^{2+}	1053.59^{2+}	313.22 (Thr-Ile-Val)	PAAVAGTVLNVVEAGGWVTTIV
575.3188	575.32		KEFGI
646.3559	646.36	71.04 (Ala)	AKEFGI
777.3964	777.40	131.04(Met)	MAKEFGI
963.4757	963.48	186.08 (Trp)	WMAKEFGI
1034.5128	1034.51	71.03 (Ala)	AWMAKEFGI
958.0586^{+2}	958.05^{+2}		KKGKRAVIAWMAKEFGI
1143.1694^{+2}	1143.17^{+2}	370.24 (Lys-Leu-Glu)	EIKKKGKRAVIAWMAKEFGI
1207.2169^{+2}	1207.22^{+2}	128.10 (Lys)	KEIKKKGKRAVIAWMAKEFGI
1271.2643^{+2}	1271.24^{+2}	128.04 (Lys)	KKEIKKKGKRAVIAWMAKEFGI
1327.8064^{+2}	1327.78^{+2}	113.08 (Leu)	LKKEIKKKGKRAVIAWMAKEFGI
1362.4694^{+3}	1362.47^{+3}		SLLAAAGRESIKAYLKKEIKKKGKRAVIAWMAKEFGI
1400.1641^{+3}	1400.16^{+3}	113.07 (Leu)	LSLLAAAGRESIKAYLKKEIKKKGKRAVIAWMAKEFGI
1419.1712^{+3}	1419.17^{+3}	57.03 (Gly)	GLSLLAAAGRESIKAYLKKEIKKKGKRAVIAWMAKEFGI
1438.1784^{+3}	1438.18^{+3}	57.03 (Gly)	GGLSLLAAAGRESIKAYLKKEIKKKGKRAVIAWMAKEFGI
$1467.1891^{+3}/1100.643$	$1467.19^{+3}/1100.6$	87.03 (Ser)	SGGLSLLAAAGRESIKAYLKKEIKKKGKRAVIAWMAKEFGI

6^{+4}	4^{+4}		
1486.1962^{+3}/1114.8990^{+4}	1486.19^{+3}/1114.90^{+4}	57.00 (Gly)	GSGGLSLLAAAGRESIKAYLKKEIKKKGKRAVIAWMAKEFGI
1519.2190^{+3}/1139.6661^{+4}	1519.22^{+3}/1139.64^{+4}	99.09 (Val)	VGSGGLSLLAAAGRESIKAYLKKEIKKKGKRAVIAWMAKEFGI
1542.8981^{+3}/1157.4254^{+4}	1542.90^{+3}/1157.42^{+4}	71.04 (Ala)	AVGSGGLSLLAAAGRESIKAYLKKEIKKKGKRAVIAWMAKEFGI
1576.5806^{+3}	1576.58^{+3}	101.04 (Thr)	TAVGSGGLSLLAAAGRESIKAYLKKEIKKKGKRAVIAWMAKEFGI
1614.2753^{+3}/1210.9583^{+4}	1614.28^{+3}/1210.96^{+4}	113.1 (Leu)	LTAVGSGGLSLLAAAGRESIKAYLKKEIKKKGKRAVIAWMAKEFGI
1651.9700^{+3}/1239.2293^{+4}	1651.96^{+3}/1239.23^{+4}	113.04 (Ile)	ILTAVGSGGLSLLAAAGRESIKAYLKKEIKKKGKRAVIAWMAKEFGI
1680.9807^{+3}/1260.9873^{+4}	1680.98^{+3}/1260.99^{+4}	87.06 (Ser)	SILTAVGSGGLSLLAAAGRESIKAYLKKEIKKKGKRAVIAWMAKEFGI
1714.0035^{+3}/1285.7544^{+4}	1714.00^{+3}/1285.75^{+4}	99.06 (Val)	VSILTAVGSGGLSLLAAAGRESIKAYLKKEIKKKGKRAVIAWMAKEFGI
1751.6982^{+3}/1314.0254^{+4}	1751.69^{+3}/1314.02^{+4}	113.07 (Leu)	IVSILTAVGSGGLSLLAAAGRESIKAYLKKEIKKKGKRAVIAWMAKEFGI
1785.3807$^{+3/}$1339.2874^{+4}	1785.38$^{+3/}$1339.28^{+4}	101.07 (Thr)	TIVSILTAVGSGGLSLLAAAGRESIKAYLKKEIKKKGKRAVIAWMAKEFGI
1819.0633^{+3}/1364.5493^{+4}	1819.06^{+3}/1364.55^{+4}	101.04 (Thr)	TTIVSILTAVGSGGLSLLAAAGRESIKAYLKKEIKKKGKRAVIAWMAKEFGI
1852.0861^{+3}/1389.3164^{+4}	1852.05^{+3}/1389.31^{+4}	98.97 (Val)	VTTIVSILTAVGSGGLSLLAAAGRESIKAYLKKEIKKKGKRAVIAWMAKEFGI
1435.8362^{+4}	1435.83^{+4}	186.17 (Trp)	WVTTIVSILTAVGSGGLSLLAAAGRESIKAYLKKEIKKKGKRAVIAWMAKEFGI
1450.0916^{+4}	1450.10^{+4}	57.08 (Gly)	GWVTTIVSILTAVGSGGLSLLAAAGRESIKAYLKKEIKKKGKRAVIAWMAKEFGI
1464.3469^{+4}	1464.35^{+4}	57.00 (Gly)	GGWVTTIVSILTAVGSGGLSLLAAAGRESIKAYLKKEIKKKGKRAVIAWMAKEFGI
1514.3669^{+4}	1514.37^{+4}	200.08 (Ala+Glu)	EAGGWVTTIVSILTAVGSGGLSLLAAAGRESIKAYLKKEIKKKGKRAVIAWMAKEFGI
1396.4038^{+5}	1396.40^{+5}		AVAGTVLNVVEAGGWVTTIVSILTAVGSGGLSLLAAAGRESIKAYLKKEIKKKGKRAVIAWMAKEFGI
1410.6112^{+5}	1410.61^{+5}	71.05 (Ala)	AAVAGTVLNVVEAGGWVTTIVSILTAVGSGGLSLLAAAGRESIKAYLKKEIKKKGKRAVIAWMAKEFGI
**Cyclized peptide broken in between A11 and V12**			
1124.4120^{+4}	1124.41^{+4}		GLSLLAAAGRESIKAYLKKEIKKKGKRAVIAWMAKEFGIPAA
1138.6674^{+4}	1138.67^{+4}	57.04 (Gly)	GGLSLLAAAGRESIKAYLKKEIKKKGKRAVIAWMAKEFGIPAA
1160.4254^{+4}	1160.43^{+4}	87.04 (Ser)	SGGLSLLAAAGRESIKAYLKKEIKKKGKRAVIAWMAKEFGIPAA
1174.6807^{+4}	1174.68^{+4}	57.00 (Gly)	GSGGLSLLAAAGRESIKAYLKKEIKKKGKRAVIAWMAKEFGIPAA
1199.4478^{+4}	1199.44^{+4}	99.04 (Val)	VGSGGLSLLAAAGRESIKAYLKKEIKKKGKRAVIAWMAKEFGIPAA

1217.2071^{+4}	1217.21^{+4}	71.08 (Ala)	AVGSGGLSLLAAAGRESIKAYLKKEIKKKGKRAVIAWMAKEFGIPAA
1270.7401^{+4}	1270.74^{+4}	214.12 (Thr-Leu)	LTAVGSGGLSLLAAAGRESIKAYLKKEIKKKGKRAVIAWMAKEFGIPAA
1299.0111^{+4}	1299.01^{+4}	113.08 (Ile)	ILTAVGSGGLSLLAAAGRESIKAYLKKEIKKKGKRAVIAWMAKEFGIPAA
1320.7691^{+4}	1320.77^{+4}	87.04 (Ser)	SILTAVGSGGLSLLAAAGRESIKAYLKKEIKKKGKRAVIAWMAKEFGIPAA
1345.5362^{+4}	1345.53^{+4}	99.04 (Val)	VSILTAVGSGGLSLLAAAGRESIKAYLKKEIKKKGKRAVIAWMAKEFGIPAA
1373.8072^{+4}	1373.80^{+4}	113.08 (Leu)	IVSILTAVGSGGLSLLAAAGRESIKAYLKKEIKKKGKRAVIAWMAKEFGIPAA
1399.0691^{+4}	1399.07^{+4}	101.08 (Thr)	TIVSILTAVGSGGLSLLAAAGRESIKAYLKKEIKKKGKRAVIAWMAKEFGIPAA
1424.3310^{+4}	1424.33^{+4}	101.04 (Thr)	TTIVSILTAVGSGGLSLLAAAGRESIKAYLKKEIKKKGKRAVIAWMAKEFGIPAA
1449.0981^{+4}	1449.10^{+4}	99.08 (Val)	VTTIVSILTAVGSGGLSLLAAAGRESIKAYLKKEIKKKGKRAVIAWMAKEFGIPAA
**Cyclized peptide broken in between V19 and V20**			
1327.0270^{+4}	1327.02^{+4}		GGLSLLAAAGRESIKAYLKKEIKKKGKRAVIAWMAKEFGIPAAVAGTVLNV
1348.7850^{+4}	1348.78^{+4}	87.04 (Ser)	SGGLSLLAAAGRESIKAYLKKEIKKKGKRAVIAWMAKEFGIPAAVAGTVLNV
1363.0404^{+4}	1363.04^{+4}	57.04 (Gly)	GSGGLSLLAAAGRESIKAYLKKEIKKKGKRAVIAWMAKEFGIPAAVAGTVLNV
1387.8075^{+4}	1387.80^{+4}	99.04 (Val)	VGSGGLSLLAAAGRESIKAYLKKEIKKKGKRAVIAWMAKEFGIPAAVAGTVLNV
1405.5667^{+4}	1405.56^{+4}	71.04 (Ala)	AVGSGGLSLLAAAGRESIKAYLKKEIKKKGKRAVIAWMAKEFGIPAAVAGTVLNV
1430.8287^{+4}	1430.82^{+4}	101.04 (Thr)	TAVGSGGLSLLAAAGRESIKAYLKKEIKKKGKRAVIAWMAKEFGIPAAVAGTVLNV
**Cyclized peptide broken in between V12 and A13**			
556.3089	556.31		AGTVLN
655.3774	655.38	99.07 (Val)	AGTVLNV
754.4458	754.45	99.07 (Val)	AGTVLNVV
883.4884	883.49	129.04 (Glu)	AGTVLNVVE
954.5255	954.53	71.04 (Ala)	AGTVLNVVEA
1011.5469	1011.55	57.02 (Gly)	AGTVLNVVEAG
1106.6527^{+4}	1106.65^{+4}		SLLAAAGRESIKAYLKKEIKKKGKRAVIAWMAKEFGIPAAV
1134.9237^{+4}	1134.91^{+4}	113.04 (Ala)	LSLLAAAGRESIKAYLKKEIKKKGKRAVIAWMAKEFGIPAAV
1531.9030^{+3}	1531.89^{+3}	57.03 (Gly)	GLSLLAAAGRESIKAYLKKEIKKKGKRAVIAWMAKEFGIPAAV
1163.4345^{+4}/1550.9102^{+3}	1163.43^{+4}/1550.91^{+3}	57.05 (Gly)	GGLSLLAAAGRESIKAYLKKEIKKKGKRAVIAWMAKEFGIPAAV
1199.4478^{+4}/1598.9280^{+3}	1199.45^{+4}/1598.93^{+3}	144.08 (Ser-Gly)	GSGGLSLLAAAGRESIKAYLKKEIKKKGKRAVIAWMAKEFGIPAAV
1224.2149^{+4}/1631.9508^{+3}	1224.22^{+4}/1631.95^{+3}	99.08 (Val)	VGSGGLSLLAAAGRESIKAYLKKEIKKKGKRAVIAWMAKEFGIPAAV
1241.9742^{+4}/1655.6299^{+3}	1241.97^{+4}/1655.63^{+3}	71.00 (Ala)	AVGSGGLSLLAAAGRESIKAYLKKEIKKKGKRAVIAWMAKEFGIPAAV
1267.2361^{+4}/1689.3124^{+3}	1267.24^{+4}/1689.31^{+3}	101.08 (Thr)	TAVGSGGLSLLAAAGRESIKAYLKKEIKKKGKRAVIAWMAKEFGIPAAV
1295.5072^{+4}/1727.007	1295.50^{+4}/1727.0	113.04 (Leu)	LTAVGSGGLSLLAAAGRESIKAYLKKEIKKKGKRAVIAWMAKEFGIPAAV

1^{+3}	1^{+3}		
1323.7782^{+4}	1323.77^{+4}	113.08 (Ile)	ILTAVGSGGLSLLAAAGRESIKAYLKKEIKKKGKRAVIAWMAKEFGIPAAV
1345.5362^{+4}/1793.7125^{+3}	1345.53^{+4}/1793.70^{+3}	87.04 (Ser)	SILTAVGSGGLSLLAAAGRESIKAYLKKEIKKKGKRAVIAWMAKEFGIPAAV
1370.3033^{+4}	1370.30^{+4}	99.08 (Val)	VSILTAVGSGGLSLLAAAGRESIKAYLKKEIKKKGKRAVIAWMAKEFGIPAAV
1398.5743^{+4}	1398.56^{+4}	113.04 (Ile)	IVSILTAVGSGGLSLLAAAGRESIKAYLKKEIKKKGKRAVIAWMAKEFGIPAAV
1423.8362^{+4}	1423.83^{+4}	101.08 (Thr)	TIVSILTAVGSGGLSLLAAAGRESIKAYLKKEIKKKGKRAVIAWMAKEFGIPAAV
1449.0981^{+4}	1449.10^{+4}	101.08 (Thr)	TTIVSILTAVGSGGLSLLAAAGRESIKAYLKKEIKKKGKRAVIAWMAKEFGIPAAV
1473.8652^{+4}	1473.86^{+4}	99.04 (Val)	VTTIVSILTAVGSGGLSLLAAAGRESIKAYLKKEIKKKGKRAVIAWMAKEFGIPAAV
1534.6404^{+4}	1534.64^{+4}	243.12 (Trp-Gly)	GWVTTIVSILTAVGSGGLSLLAAAGRESIKAYLKKEIKKKGKRAVIAWMAKEFGIPAAV
